# Memory Decline and Depression Onset in U.S. and European Older Adults

**DOI:** 10.1177/0898264318813019

**Published:** 2018-11-23

**Authors:** Rebecca Bendayan, Amanda Kelly, Scott M. Hofer, Andrea M. Piccinin, Graciela Muniz-Terrera

**Affiliations:** 1 MRC Unit for Lifelong Health and Ageing at UCL, UK; 2 Department Biostatistics and Health Informatics, Institute of Psychiatry, Psychology & Neuroscience (IoPPN), King’s College London, UK; 3 Department of Psychology, University of Victoria, British Columbia, Canada; 4 Centre for Dementia Prevention, University of Edinburgh, Scotland

**Keywords:** cognitive decline, depressive symptoms, HRS, replication, SHARE

## Abstract

**Objectives:** We explore the association between different patterns of change in depressive symptoms and memory trajectories in US and European Mediterranean (Spain, France, Italy, and Israel) and non-Mediterranean (Sweden, Denmark, Netherlands, Germany, Belgium, Switzerland, and Austria) older adults. **Methods:** Samples consisted of 3,466 participants from the Health Retirement Study (HRS) and 3,940 participants from the Survey of Health, Aging and Retirement (SHARE). Individuals were grouped as follows: non-case depression (NO DEP), persistent depression (DEP), depression onset (ONSET), depression recovery (RECOV), and fluctuating (FLUCT). Memory was measured using immediate and delayed recall tests. Linear mixed models were used. **Results:** DEP and RECOV had significantly lower baseline memory scores compared to NO DEP, at intercept level. At slope level, ONSET had a significantly faster decline in both tasks compared to NO DEP. **Discussion:** Cross-cohort robust and consistent new empirical evidence on the association between depression onset and faster decline in memory scores is provided.

## Introduction

The association between cognitive function and depression in older adults has been extensively studied (Christensen, Griffiths, MacKinnon, & Jacomb, 1997; Jorm, 2000; Rock, Roiser, Riedel, & Blackwell, 2014). Cross-sectional studies have found that depressive symptomatology is associated with poor cognitive function (Brevik, Eikeland, & Lundervold, 2013; Fuhrer et al., 1992; Gale et al., 2011; Langa et al., 2009; Scherr et al., 1988), and longitudinal studies have also found that higher scores in depression at baseline are associated with a steeper decline in cognitive function (Gale, Allerhand, & Deary, 2012; Raji, Reyes-Ortiz, Kuo, Markides & Ottenbacher, 2007; Wilson et al., 2002; Zahodne, Stern, & Manly, 2014). However, differences by age and cognitive measure have been found. For example, Gale et al. (2012) found this association only in people aged 60 to 80 years, and Wilson et al. (2002) found that depressive symptoms were only associated with a faster decline in episodic memory and visuospatial ability, but not in perceptual speed, or working or semantic memory.

Most of the aforementioned studies include depression at baseline, assuming that depressive symptomatology in older adults follows a stable pattern. However, a number of studies have provided evidence regarding changes in depression over time (Cui, Lyness, Tang, Tu, & Conwell, 2008; Licht-Strunk et al., 2009; Van Orden, Chen, O’ Riley, & Conwell, 2015), though few have accounted for changes in depression (Dufouil, Fuhrer, Dartigues, & Alpérovitch, 1996; Houde, Bergman, Whitehead, & Chertkow, 2008; Köhler et al., 2010; Paterniti, Verdier-Taillefer, Dufouil, & Alpérovitch, 2002), and these studies have mainly compared patterns of persistent and not persistent depression. Some found that the association between depression and cognitive decline can only be found when depression symptoms are persistent (Houde et al., 2008; Paterniti et al., 2002), whereas Köhler et al. (2010) found a significant association between cognitive decline and depression at baseline that becomes stronger when depressive symptoms are persistent. Dufouil et al. (1996) also explored the association between change in cognitive scores and change in depression over 3 years and suggested that cognitive decline was associated with the concomitant level of depressive symptomatology independent of depressive symptoms at baseline.

Within this context, the general aim of the present study is to explore the association between different patterns of change in depressive symptoms and trajectories of immediate and delayed recall in U.S. and European older adults.

## Methods

### Sample

The U.S. sample consisted of a subsample of 3,466 respondents (1,397 men and 2,069 women) of the Health Retirement Study (HRS) and the European sample consisted of a subsample of 3,940 respondents (1,808 men and 2,132 women) of the Survey of Health, Aging and Retirement (SHARE). Both studies are biannual, longitudinal, and nationally representative surveys that focus on adults aged 50 and over. Data from five consecutive waves of each study have been used: Wave 4 (1998) to Wave 8 (2006) of HRS, and Wave 1 (2004) to Wave 5 (2013) of SHARE (Wave 3 on SHARE was not included as it did not have available data on the relevant variables for the present study). Wave 4 of HRS was selected as “baseline” as 1998 is when HRS and AHEAD were fully integrated and when HRS assessment procedure matched other sister studies as SHARE. Considering previous research (Castro-Costa et al., 2007; Litwin, 2009), the countries were classified in two groups: Mediterranean (Spain, France, Italy, and Israel) and non-Mediterranean (Sweden, Denmark, the Netherlands, Germany, Belgium, Switzerland, and Austria).

For the purpose of this study, the first wave considered will be labeled as baseline and the subsequent ones follow-ups. Only respondents who were between 65 and 75 years old at the first wave were considered (baseline) and who had at least 3 waves of complete data in depressive symptoms and immediate and delayed recall were included, according to Singer and Willett (2003) assumptions, to capture change.

### Measures

#### Memory

Both HRS and SHARE assessed memory using tests of immediate and delayed recall of 10 common nouns. Respondents were asked to recall as many words as possible immediately after the list was read and after a short delay of 5 min, during which they completed other cognitive tests. In both studies, different randomly assigned word lists were used at each occasion. Total scores are the number of words recalled in each test and range between 0 and 10, with higher scores indicative of better memory.

#### Depressive symptoms

In HRS, depressive symptoms were assessed by the 8-item version of the Center For Epidemiologic Studies Depression Scale (CES-D), which is a questionnaire developed as a depressive symptoms screening scale for epidemiological investigation (Radloff, 1977; Turvey, Wallace, & Herzog, 1999). Its validity and reliability has been found to be comparable to the 20-item version of this scale (Karim, Weisz, Bibi, & Rehman, 2015), and a cut-off of 4 or more symptoms on the 8-item scale to determine case-level depression (i.e., clinically significant depressive symptoms) has been extensively used in aging research (e.g., Ní Mhaolain et al., 2012; Péquignot et al., 2016; Pun, Manjourides, & Suh, 2016). In SHARE, depression was assessed by the EURO-D, which was originally developed to compare symptoms of depression in 11 European centers (Prince, Beekman et al., 1999). Its items are derived from the Geriatric Mental State examination (GMS; Copeland, Dewey, & Griffiths-Jones, 1986) and cover 12 symptom domains: depressed mood, pessimism, suicidality, guilt, sleep, interest, irritability, appetite, fatigue, concentration, enjoyment, and tearfulness. Each item is scored 0 (*symptom not present*) or 1 (*symptom present*), and item scores are summed to produce a scale with a minimum score of zero and a maximum of 12. The psychometric properties of the EURO-D have been extensively investigated and criterion validity demonstrated in the cross-cultural context. Criterion validity for this measure was demonstrated with an optimal cut-off point of a score of 4 or above against a variety of criteria for clinically significant depression (Prince, Reischies et al., 1999). Previous research has found that CES-D and EURO-D are comparable measures of depression when examining their association with other health outcomes (Courtin, Knapp, Grundy, & Avendano-Pabon, 2015).

To identify the different patterns of change described in previous research (Cable, Chandola, Aida, Sekine, & Netuveli, 2017; Cui et al., 2008; Licht-Strunk et al., 2009; Van Orden et al., 2015), we followed the steps described next. First, the scores in both scales were coded in two categories, using the cut-off of 4 or more symptoms on the scale to determine case-level depression. Then, changes in depressive symptoms were grouped into 5 patterns: (a) non case depression (NO DEP): less than four clinically significant depressive symptoms at every wave; (b) persistent depression (DEP): four or more clinically significant depressive symptoms at every wave; (c) depression onset (ONSET): increase of clinically significant depressive symptoms over time (from less than four clinically significant depressive symptoms to four or more); (d) depression recovery (RECOV): decrease of clinically significant depressive symptoms over time (from four or more clinically significant depressive symptoms to less than four); and (e) fluctuating pattern (FLUCT): clinically significant depressive symptoms fluctuating without a trend.

#### Covariates

Baseline age, sex, education and self-rated health (SRH). Education was coded in three categories (9 years or less, 10-13 years, and 14 or more years). SRH reflects the general perception of an individual’s health status, and it is one of the most inclusive and informative indicators of health status (Jylhä, 2009). SRH, originally a 5 category variable (excellent, very good, good, fair, and poor), was recoded into two categories: (a) excellent, very good, and good and (b) fair or poor. This dichotomization has been used in previous research (Diehr, Thielke, Newman, Hirsch, & Tracy, 2013; Leinonen, Heikkinen, & Jylhä, 2002).

### Statistical Analyses

Preliminary descriptive and exploratory analysis examined differences at baseline between groups within each study. Differences were examined using chi-square and Kruskal–Wallis tests. To examine the trajectories of immediate and delayed recall over time, in each group, linear mixed models with random coefficients (Laird & Ware, 1982) were estimated with SAS Proc Mixed (Littell, Milliken, Stroup, & Wolfinger, 1996). Linear and quadratic conditional models were fitted, adjusted for all the covariates considered. Dichotomous covariates were effect-coded, baseline age was centered at 65 years, and only significant estimates were included in the final models. Sensitivity analyses were performed including baseline depression.

## Results

### Descriptive and Exploratory Analyses

Descriptive statistics for depressive symptoms, immediate and delayed recall and covariates at baseline in the study samples are shown in Table 1 and descriptive statistics for each pattern of change in depression scores in both studies are shown in Table 2.

**Table 1.** Descriptive Statistics at Baseline in HRS (*N* = 3,466) and SHARE Mediterranean (*N* = 1,587), and Non-Mediterranean Countries (*N* = 2,353).

		SHARE	
	HRS	Mediterranean countries	Non-Mediterranean countries
Baseline age	69.42 (3.19)	69.45 (3.05)	69.34 (3.11)
Sex (female)	2,069 (59.7%)	882 (55.6%)	1,250 (53.1%)
Education			
9 years or less	549 (15.8%)	920 (61.3%)	841 (38.8%)
10-13	1,816 (52.4%)	306 (20.4%)	753 (34.7%)
14+	1,101 (31.8%)	232 (15.4%)	534 (24.6%)
Immediate recall	5.74 (1.64)	3.95 (1.63)	5.12 (1.60)
Delayed recall	4.62 (2.04)	2.55 (1.68)	3.65 (1.90)
Depressive symptoms			
Clinically sign ⩾ 4	405 (11.7%)	510 (32.1%)	377 (16.0%)
SRH			
Excellent/Very Good/Good	2,695 (77.8%)	1,231 (77.9%)	2,083 (88.6%)

*Note.* HRS = Health Retirement Study; SHARE = Survey of Health, Aging and Retirement; SRH = Self-rated health.

**Table 2.** Descriptive Statistics for Immediate and Delayed Recall Tests at Each Follow-Up for Each Pattern of Change in Depressive Symptoms in HRS, SHARE Mediterranean Countries, and SHARE Mediterranean Countries.

	HRS					SHARE—Mediterranean					SHARE—Non-mediterranean				
	NO DEP	FLUCT	ONSET	RECOV	DEP	NO DEP	FLUCT	ONSET	RECOV	DEP	NO DEP	FLUCT	ONSET	RECOV	DEP
*n*	2,377 (68.6%)	624 (18%)	225 (6.5%)	167 (4.8%)	73 (2.1%)	661 (41.7%)	298 (18.8%)	251 (15.8%)	203 (12.8%)	174 (11.0%)	1,422 (60.4%)	339 (14.4%)	305 (13.0%)	175 (7.4%)	112 (4.8%)
Immediate recall baseline	5.86 (1.61)	5.74 (1.63)	5.65 (1.76)	5.51 (1.66)	4.72 (1.60)	4.28 (1.64)	3.68 (1.58)	3.97 (1.52)	3.72 (1.60)	3.40 (1.58)	5.23 (1.57)	5.01 (1.60)	5.08 (1.57)	4.70 (1.68)	4.79 (1.80)
First follow-up	5.54 (1.63)	5.28 (1.64)	5.40 (1.63)	5.29 (1.62)	4.60 (1.49)	4.34 (1.65)	3.78 (1.59)	4.13 (1.69)	3.92 (1.59)	3.25 (1.61)	5.27 (1.60)	5.02 (1.71)	5.07 (1.65)	4.68 (1.68)	4.90 (1.48)
Second follow-up	5.40 (1.63)	5.15 (1.63)	5.11 (1.62)	5.21 (1.56)	4.52 (1.65)										
Third follow-up	5.22 (1.54)	4.85 (1.63)	4.86 (1.46)	4.81 (1.64)	4.30 (1.58)	4.21 (1.66)	3.99 (1.77)	3.83 (1.79)	3.72 (1.69)	3.21 (1.89)	4.96 (1.55)	4.83 (1.70)	4.47 (1.90)	4.63 (1.76)	4.59 (1.78)
Fourth follow-up	5.00 (1.56)	4.57 (1.62)	4.47 (1.74)	4.64 (1.60)	3.98 (1.51)	4.42 (1.74)	3.74 (1.66)	3.63 (1.83)	4.04 (1.80)	3.26 (1.77)	4.86 (1.62)	4.80 (1.66)	4.61 (1.69)	4.39 (1.85)	4.27 (1.92)
Delayed recall baseline	4.74 (2.01)	4.39 (2.06)	4.57 (2.16)	4.39 (2.07)	3.36 (1.85)	2.80 (1.74)	2.44 (1.60)	2.61 (1.65)	2.26 (1.57)	2.05 (1.60)	3.79 (1.87)	3.58 (1.93)	3.46 (2.00)	3.23 (1.81)	3.17 (1.83)
First follow-up	4.51 (1.96)	4.18 (2.04)	4.39 (1.99)	4.27 (2.07)	3.70 (1.72)	2.93 (1.79)	2.35 (1.73)	2.70 (1.70)	2.45 (1.59)	2.04 (1.76)	3.88 (1.85)	3.55 (1.98)	3.61 (1.96)	3.37 (1.97)	3.37 (1.77)
Second follow-up	4.37 (1.97)	4.04 (2.09)	4.19 (1.92)	4.05 (2.05)	3.38 (2.05)										
Third follow-up	4.06 (1.88)	3.71 (1.97)	3.52 (2.00)	3.80 (1.97)	2.94 (2.04)	2.71 (1.91)	2.44 (1.90)	2.42 (1.99)	2.27 (1.75)	1.70 (1.83)	3.68 (1.98)	3.45 (2.10)	3.20 (2.08)	3.25 (2.25)	3.11 (2.17)
Fourth follow-up	3.81 (1.91)	3.31 (1.97)	3.40 (2.24)	3.56 (2.06)	2.52(1.87)	2.98 (1.92)	2.15 (1.88)	2.15 (1.81)	2.58 (1.85)	1.72 (1.67)	3.45 (2.09)	3.38 (2.07)	2.98 (2.03)	3.19 (2.23)	2.70 (2.06)
Covariates															
Age baseline	69.41 (3.20)	69.52 (3.22)	69.58 (2.92)	69.30 (3.38)	68.75 (3.04)	69.06 (3.07)	69.30 (3.24)	69.59 (3.31)	69.35 (3.24)	70.03 (3.13)	69.42 (3.16)	69.48 (3.20)	69.61 (3.04)	69.69 (3.27)	69.21 (3.30)
Female	53.7%	71.8%	72.9%	71.9%	82.2%	272 (41.1%)	190 (63.8%)	151 (60.2%)	130 (64.0%)	139 (79.9%)	647 (45.5%)	212 (62.5%)	186 (61.0%)	120 (68.6%)	85 (75.9%)
Education															
9 years or less	287 (12.1%)	158 (25.3%)	38 (16.9%)	33 (19.8%)	33 (45.2%)	319 (51.5%)	207 (72.1%)	149 (62.1%)	121 (62.4%)	124 (77.0%)	483 (37.2%)	130 (40.5%)	105 (37.4%)	82 (50.0%)	41 (39.4%)
10-13 years	1,218 (51.2%)	336 (53.8%)	133 (7.3%)	93 (55.7%)	36 (49.3%)	146 (23.5%)	47 (16.4%)	48 (20.0%)	41 (21.1%)	24 (14.9%)	448 (34.5%)	119 (37.1%)	104 (37.0%)	46 (28.0%)	36 (34.6%)
14+ years	872 (36.7%)	130 (20.8%)	54 (24%)	41 (24.6%)	4 (5.5%)	141 (22.7%)	24 (8.4%)	32 (13.3%)	28 (14.4%)	7 (4.3%)	346 (26.6%)	67 (20.9%)	63 (22.4%)	33 (20.1%)	25 (24.0%)
SRH e/vg/g	85.1%	61.7%	68%	68.5%	28.8%	585 (88.6%)	220 (74.1%)	200 (90.0%)	137 (68.2%)	89 (51.4%)	1,349 (94.9%)	276 (81.4%)	250 (82.0%)	133 (76.4%)	75 (67.0%)

*Note.* HRS = Health Retirement Study; SHARE = Survey of Health, Aging and Retirement; NO DEP = non case depression; DEP = persistent depression; ONSET = depression onset; RECOV = depression recovery; FLUCT = fluctuating pattern; SRH e/vg/g = Excellent, very good, and good self-rated health.

Despite the variation of prevalence rates for each of the observed patterns of change in depressive symptoms in HRS and SHARE Mediterranean and non-Mediterranean countries, when we order the prevalence from highest to lowest, we find the same order (Table 2). Most of the participants reported less than four clinically significant depressive symptoms across all waves (NO DEP), followed by clinically significant depressive symptoms fluctuating without a trend (FLUCT), increase of clinically significant depressive symptoms over time (ONSET), decrease of clinically significant depressive symptoms from wave over time (RECOV), and, least reported, four or more clinically significant depressive symptoms across all waves (DEP). When immediate and delayed recall scores were compared between the groups in each study, overall differences were found between all the groups at each follow-up. Differences between groups in each study were also found for baseline covariates (except for baseline age in HRS and SHARE-Mediterranean countries).

### Longitudinal Analyses

In HRS and SHARE Mediterranean and non-Mediterranean countries, we found an association between the pattern of change in depressive symptoms and immediate and delayed recall scores at intercept level (Tables 3 and 4). Individuals who reported less than four clinically significant depressive symptoms across all waves (NO DEP) showed higher immediate and delayed memory scores compared to all the other groups except those reporting an increase of clinically significant depressive symptoms over time (ONSET). Younger individuals at baseline with higher levels of education reported higher baseline scores in immediate and delayed recall in both studies. Women in HRS and SHARE-Non Mediterranean countries also had higher baseline cognitive scores than men, but no sex differences were found for SHARE Mediterranean participants. Self-reported health at baseline was only associated with memory in HRS, with individuals who perceived their health as excellent, very good, or good at baseline scoring higher on memory at baseline.

**Table 3.** Parameter Estimates, Standard Errors, and CIs From Immediate Recall Linear Mixed Models Adjusted for Baseline Age, Sex, Education, and SRH at Baseline in HRS and SHARE Mediterranean and Non-Mediterranean Countries.

	HRS		SHARE-Mediterranean		SHARE- Non mediterranean	
	Estimate (*SE*)	CI	Estimate (*SE*)	CI	Estimate (*SE*)	CI
Intercept	5.25 (0.08)***	[5.07, 5.42]	3.96 (0.08)***	[3.78, 4.13]	5.07 (0.07)***	[4.93, 5.21]
Slope_linear	−0.09 (0.005)***	[−0.10, −0.08]	0.07 (0.03)**	[0.01, 0.13]	−0.05 (0.006)***	[−0.06, −0.03]
Slope_quadratic			−0.005 (0.002)*	[−0.01, −0.0001]		
Age_baseline	−0.07 (0.005)***	[−0.08, −0.06]	−0.07 (0.009)***	[−0.09, −0.05]	−0.06 (0.008)***	[0.39, 0.60]
Female	0.62 (0.05)***	[0.51, 0.72]			0.50 (0.05)***	[0.39, 0.60]
Education (ref 9 years or less)						
10-13	0.80 (0.05)***	[0.70, 0.91]	0.93 (0.07)***	[0.79, 1.08]	0.29 (0.05)***	[0.17, 0.41]
14+	1.28 (0.05)***	[1.17, 1.39]	1.36 (0.08)***	[1.19, 1.53]	0.77 (0.06)***	[0.64, 0.90]
SRH e/vg/g_baseline	0.20 (0.04)***	[0.11, 0.29]				
Depressive symptoms (ref NO DEP)						
DEP	−0.77 (0.18)***	[–1.12, −0.41]	−0.55 (0.12)***	[−0.81, −0.30]	−0.45 (0.15)**	[−0.76, −0.14]
ONSET	−0.06 (0.10)	[−0.27, 0.13]	0.09 (0.11)	[−0.12, 0.31]	−0.12 (0.10)	[−0.32, 0.07]
RECOV	−0.24 (0.12)*	[−0.47, −0.005]	−0.24 (0.12)*	[−0.49, −0.007]	−0.67 (0.13)***	[−0.93, −0.40]
FLUCT	−0.13 (0.03)*	[−0.27, −0.001]	−0.33 (0.10)***	[−0.54, −0.12]	−0.31 (0.09)**	[−0.50, −0.12]
Slope linear						
Female	0.01 (0.007)*	[0.001, 0.02]				
Depressive symptoms (ref NO DEP)						
DEP	0.02 (0.02)	[−0.02, 0.07]	−0.02 (0.01)	[−0.06, 0.01]	−0.02 (0.02)	[−0.06, 0.02]
ONSET	−0.03 (0.01)**	[−0.06, −0.01]	−0.06 (0.01)***	[−0.10, −0.03]	−0.03 (0.01)**	[−0.07, −0.09]
RECOV	−0.007 (0.01)	[−0.04, 0.02]	−0.01 (0.01)	[−0.04, 0.02]	0.04 (0.02)*	[0.004, 0.08]
FLUCT	−0.01 (0.0009)	[−0.02, 0.007]	0.004 (.01)	[−0.02, 0.03]	0.01 (0.01)	[−0.008, 0.04]
Variances						
Intercept	1.08 (0.04)***		0.77 (0.08)***		0.94 (0.07)***	
Slope linear	0.008 (0.001)***		0.005 (0.002)**		0.008 (0.001)***	
Residual	1.37 (0.01)***		1.56 (0.04)***		1.47 (0.03)***	
Fit statistics						
−2LL	60,021.4		19,386.7		27,040.8	
BIC	60,054		19,416		27,071.2	

*Note.* CI = confidence interval; HRS = Health Retirement Study; SHARE = Survey of Health, Aging and Retirement; NO DEP = non case depression; DEP = persistent depression; ONSET = depression onset; RECOV = depression recovery; FLUCT = fluctuating pattern; SRH e/vg/g = excellent, very good and good self-rated health.

* *p* < .05. **p < .01. ***p < .001.

**Table 4.** Parameter Estimates, Standard Errors, and CIs From Delayed Recall Linear Mixed Models Adjusted for Baseline Age, Sex, Education and SRH at Baseline in HRS and SHARE Mediterranean and Non-Mediterranean Countries.

	HRS		SHARE-Mediterranean		SHARE-non Mediterranean	
	Estimate (*SE*)	CI	Estimate (*SE*)	CI	Estimate (*SE*)	CI
Intercept	4.20 (0.12)***	[3.96, 4.44]	2.56 (0.09)***	[2.37, 2.74]	3.46 (0.09)***	[3.27, 3.65]
Slope_linear	−0.07 (0.01)***	[−0.10, −0.05]	0.08 (0.03)**	[0.01, 0.14]	0.08 (0.02)**	[0.02, 0.13]
Slope_quadratic			−0.006 (0.003)*	[−0.01, −0.005]	−0.01 (0.02)***	[−0.01, −0.07]
Age_baseline	−0.10 (0.007)***	[−0.11, −0.08]	−0.08 (0.009)***	[−0.10, −0.06]	−0.07 (0.01)***	[−0.09, −0.05]
Female	0.76 (0.06)***	[0.64, 0.89]			0.61 (0.06)***	[0.47, 0.74]
Education (ref 9 years or less)						
10-13	0.87 (0.05)***	[0.69, 1.05]	0.86 (0.07)***	[0.71, 1.02]	0.24 (0.07)**	[0.09, 0.39]
14+	1.50 (0.09)***	[1.30, 1.69]	1.36 (0.09)***	[1.18, 1.54]	0.78 (0.08)***	[0.61, 0.94]
SRH_baseline	0.23 (0.08)***	[0.07, 0.39]				
Depressive symptoms (ref NO DEP)						
DEP	−0.76 (0.22)***	[–1.21, −0.32]	−0.38 (0.13)***	[−0.65, −0.11]	−0.69 (0.19)***	[–1.08, −0.31]
ONSET	−0.01 (0.13)	[−0.27, 0.23]	0.16 (0.11)	[−0.06, 0.39]	−0.17 (0.12)	[−0.42, 0.07]
RECOV	−0.28 (0.15)*	[−0.57, 0.009]	−0.38 (0.13)**	[−0.64, −0.11]	−0.61 (0.16)***	[−0.94, −0.29]
FLUCT	−0.14 (0.08)	[−0.31, −0.02]	−0.08 (0.11)	[−0.30, 0.13]	−0.39 (0.11)**	[−0.62, −0.15]
Slope Linear						
Female	0.03 (0.009)***	[0.01, 0.05]				
Education (ref 9 years or less)						
10-13	−0.01 (0.01)	[−0.03, 0.01]				
14+	−0.03 (0.01)**	[−0.06, −0.005]				
Depressive symptoms (ref NO DEP)						
DEP	−0.003 (0.03)	[−0.06, 0.05]	−0.05 (0.02)**	[−0.09, −0.01]	−0.03 (0.02)	[−0.09, 0.02]
ONSET	−0.04 (0.01)*	[−0.07, −0.004]	−0.07 (0.01)***	[−0.10, −0.03]	−0.04 (0.01)**	[−0.08, −0.01]
RECOV	0.009 (0.02)	[−0.03, 0.05]	0.009 (0.02)	[−0.03, 0.05]	0.04 (0.02)	[−0.007, −0.08]
FLUCT	−0.01 (0.01)	[−0.03, 0.01]	−0.04 (0.01)**	[−0.07, −0.01]	0.01 (0.01)	[−0.01, 0.05]
Variances						
Intercept	1.70 (0.08)***		1.00 (0.10)***		1.80 (0.011)***	
Slope linear	0.01 (0.001)***		0.01 (0.002)***		0.02 (0.002)***	
Residual	2.08 (0.02)***		1.66 (0.04)***			
Fit statistics						
–2LL	67,368.7		19,935.6		29,421	
BIC	67,401.3		19,964.9		29,451.3	

*Note.* CI = confidence interval; HRS = Health Retirement Study; SHARE = Survey of Health, Aging and Retirement; NO DEP = non case depression; DEP = persistent depression; ONSET = depression onset; RECOV = depression recovery; FLUCT = fluctuating pattern; SRH e/vg/g = excellent, very good and good self-rated health.

* *p* < .05. **p < .01. ***p < .001.

With regard to change over time, a significant overall linear decline in immediate recall was found in HRS and SHARE-Non Mediterranean samples. The immediate recall trajectories in SHARE Mediterranean sample were better described by a quadratic model. Individuals who reported a pattern of increasing clinically significant depressive symptoms over time (ONSET) showed a slightly faster decline in their immediate recall scores when compared to those who had reported less than four clinically significant depressive symptoms across all waves (NO DEP) in all the samples. Moreover, in the SHARE non-Mediterranean sample, individuals whose clinically significant depressive symptoms decreased over time (RECOV) also showed a slower decline in immediate recall compared to NO DEP. No associations with rate of change were found for any of the covariates except that HRS women who showed a slower decline over time compared to men.

For delayed recall, linear models were appropriate for the trajectories in HRS, whereas quadratic models showed a better fit in SHARE. Similar to immediate recall, individuals reporting a pattern of increasing clinically significant depressive symptoms over time (ONSET) showed a slightly faster decline in their delayed recall scores when compared to those who had reported less than four clinically significant depressive symptoms across all waves (NO DEP) in HRS and SHARE Mediterranean and non-Mediterranean samples. In addition, those who endorsed depressive symptoms persistently over time (DEP) and those who had a fluctuating pattern with no trend (FLUCT) also showed a slightly faster decline in their delayed recall scores when compared with NO DEP. Differences in the rate of change were not found for any of the covariates except sex and education level in HRS. Consistent results were found for sensitivity analyses which included baseline depression in the model.

## Discussion

The main aim of the present study was to explore the association between different patterns of change in depression and memory trajectories in U.S. and European participants of the HRS and SHARE, respectively. This study is the first, to our knowledge, that goes beyond patterns of persistent versus not persistent depression described in previous literature on cognitive decline (Houde et al., 2008; Köhler et al., 2010; Paterniti et al., 2002). Specifically, we included patterns of change describing depression onset or recovery over time accounting for the fact that depression cannot be assumed to be stable over time. Study participants were classified into five patterns of change in depression scores and showed the same distribution in HRS and SHARE Mediterranean and non-Mediterranean countries. Most of the participants reported less than four clinically significant depressive symptoms across all waves, followed by clinically significant depressive symptoms fluctuating without a trend, increase of clinically significant depressive symptoms over time, decrease of clinically significant depressive symptoms from wave over time, and the least reported four or more clinically significant depressive symptoms across all waves. In addition, for immediate recall, individuals who had persistent depression, recovery, or a fluctuating pattern had lower baseline scores than those with no depression across all the waves considered. For delayed recall, individuals who had persistent depression or recovery had lower baseline scores than those with no depression across all the waves considered.

With regard to the rates of change in immediate and delayed recall scores and their association with the above mentioned patterns of change in depression, it was found, consistently across studies, that individuals who had an onset of depression across waves showed a faster decline in cognitive performance compared to those with no depression over time. It should be noted that although the individuals identified with persistent depression over time also showed, in general, a faster decline when compared with those individuals with no depression, the estimate was not significant.

Our results cannot be directly compared with previous research that only considered persistent and not persistent patterns with shorter follow-ups of 2 or 3 years (Dufouil et al., 1996; Houde et al., 2008), 4 years (Paterniti et al., 2002), or 6 years (Köhler et al., 2010). However, they are consistent with those reported by Köhler et al. (2010) who found that the associations of depressive symptoms with cognitive decline were stronger for those individuals with persistent depression, defined as high depressive symptomatology at baseline and at least one follow-up visit. Similarly, Paterniti et al. (2002) found that only persistent but not episodic depressive symptoms were associated with cognitive decline. These inconsistencies in the results might be associated with the cognitive measure used in their study (i.e., Mini–Mental State Examination [MMSE]) or the shorter follow-up. Furthermore, it is likely that individuals identified in the present study with a pattern of onset, recovery, or fluctuating would have been classified as persistent in studies that classified their participants only into persistent versus not persistent (Dufouil et al., 1996; Houde et al., 2008; Köhler et al., 2010; Paterniti et al., 2002). It should be noted that in our analysis those with persistent depression had consistently lower immediate and delayed recall scores at baseline compared to all the other groups. In addition, we also found that individuals who showed an onset of case level depression across waves had consistently a faster decline in immediate and delayed recall compared to those with no depression. Taking together these results, our findings could suggest that those with persistent depression (which could include long-term depression before the baseline measurement) might have begun to show decline in their memory before the baseline measurement, and, thus, the timing of depression onset might be a key factor to further our understanding of when memory decline might start to be evident. Further studies with a younger population should address the potential relationship between the timing of depression onset and the early signs of cognitive decline.

The main advantage of the present study is that its design and statistical analysis facilitates comparability across samples and future replication studies. Replication studies are essential to build scientific knowledge, and recent publications have highlighted the need to promote systematic replication efforts (Koole & Lakens, 2012; Open Science Collaboration et al., 2015), especially in longitudinal studies of aging (Hofer & Piccinin, 2009). Moreover, an integrative systematic data analysis approach was used (Hofer & Piccinin, 2009). As Hofer highlights, another possible source of variation when performing replications or comparing results between studies is the different statistical procedures followed. In the present study, the same statistical procedure was followed, reducing the possible variation due to methodological issues in three samples from two large population studies of U.S. and European older adults. Our findings provide consistent and robust evidence of the association between the patterns of change in depression and memory trajectories in older adults in the U.S. and Europe, but it would also be of great interest to perform further replications with other populations. The second main advantage of this study is that we used verbal memory measures, which have shown to be one of the strongest predictors of dementia risk in older adults (Elias et al., 2000; Howieson et al., 1997). Future studies should replicate the present including other cognitive measures, as previous research has found some differences in the association of depression with different cognitive domains (Wilson et al., 2002). The third main advantage of the present study and perhaps the most novel is the identification of patterns of change in depression beyond persistence. These were consistently found across HRS and SHARE data, and recent research in English and Japanese aging studies have identified similar patterns (Cable et al., 2017). Future research on depression should account for these patterns and explore further their association with other health outcomes. Some additional limitations derived from our efforts to make our findings replicable and comparable should be acknowledged. First, only linear and quadratic models were examined in the present study; though there might be other non-linear models which could better represent the memory trajectories in SHARE and account for potential re-test effects. Second, although SRH was used as an overall measure of health status proven to be an adequate proxy to evaluate overall health in older adults (Idler & Benyamini, 1997; Jylhä, 2009) even when compared with objective measures of health (Lima-Costa, Cesar, Chor, & Proietti, 2011; Wu et al., 2013), it would be interesting if future studies could examine the potential role of specific health conditions in this association. Third, although the sample of these studies are nationally representative, these older individuals may only represent a subset of survivors of their birth cohort (Hofer & Sliwinski, 2006) and future research comparing with later birth cohorts and data from low- and middle-income countries would be of great interest as some education inequalities seem to arise in this birth cohort. Finally, although the robustness and consistency results over three samples from representative surveys of U.S. and European Mediterranean and non-Mediterranean countries contribute to better understanding of the association between depression and cognitive performance from a public health perspective, causal relationships cannot be attributed. Previous longitudinal studies have suggested that depressive symptoms appear to predict cognitive decline but not vice-versa (Gale et al., 2012; Zahodne et al., 2014) and future research should aim to further examine the directionality of these relationships when taking into account different patterns of change in depressive symptomatology.

## Conclusion

The results of the present study not only support the well-known association between depressive symptomatology and cognitive performance in older adults, but provide new empirical evidence on the association between depression onset and faster decline in memory scores in U.S. and European adults over 65 years old. Our findings suggest that not simply duration, but the timing of onset depression, might be the key to understanding the association between depression and cognitive decline.
